# Comparative Analysis of Cytomegalovirus Gastrointestinal Disease in Immunocompetent and Immunocompromised Patients

**DOI:** 10.3390/v16030452

**Published:** 2024-03-14

**Authors:** Pai-Jui Yeh, Ren-Chin Wu, Yung-Kuan Tsou, Chien-Ming Chen, Cheng-Tang Chiu, Chien-Chang Chen, Ming-Wei Lai, Yu-Bin Pan, Puo-Hsien Le

**Affiliations:** 1 Department of Pediatric Gastroenterology, Linkou Chang Gung Memorial Hospital, Taoyuan 333, Taiwan; charlie01539@hotmail.com (P.-J.Y.); cgj2841@cgmh.org.tw (C.-C.C.); a22141@cgmh.org.tw (M.-W.L.); 2 Inflammatory Bowel Disease Center, Linkou Chang Gung Memorial Hospital, Taoyuan 333, Taiwan; renchin.wu@gmail.com (R.-C.W.); dr.cmchen@gmail.com (C.-M.C.); ctchiu@cgmh.org.tw (C.-T.C.); 3 Chang Gung Microbiota Therapy Center, Linkou Chang Gung Memorial Hospital, Taoyuan 333, Taiwan; 4 Translational Gastroenterology Unit, Nuffield Department of Medicine, University of Oxford, Oxford OX3 9DU, UK; 5 Department of Anatomic Pathology, Linkou Chang Gung Memorial Hospital, Taoyuan 333, Taiwan; 6 Department of Gastroenterology and Hepatology, Linkou Chang Gung Memorial Hospital, Taoyuan 333, Taiwan; flying@cgmh.org.tw; 7 Department of Medical Imaging and Interventions, Linkou Chang Gung Memorial Hospital, Taoyuan 333, Taiwan; 8 Taiwan Association of the Study of Small Intestinal Disease, Taoyuan 333, Taiwan; 9 Biostatistical Section, Clinical Trial Center, Chang Gung Memorial Hospital, Linkou Branch, Taoyuan 333, Taiwan; e8901145@gmail.com

**Keywords:** cytomegalovirus, gastrointestinal disease, immune status, antiviral therapy

## Abstract

Background: Cytomegalovirus (CMV) gastrointestinal (GI) diseases impact both immunocompromised and immunocompetent individuals, yet comprehensive studies highlighting the differences between these groups are lacking. Methods: In this retrospective study (January 2000 to July 2022) of 401 patients with confirmed CMV GI diseases, we categorized them based on immunological status and compared manifestations, treatments, outcomes, and prognostic factors. Results: The immunocompromised patients (n = 193) showed older age, severe illnesses, and higher comorbidity rates. GI bleeding, the predominant manifestation, occurred more in the immunocompetent group (92.6% vs. 63.6%, *p* = 0.009). Despite longer antiviral therapy, the immunocompromised patients had higher in-hospital (32.2% vs. 18.9%, *p* = 0.034) and overall mortality rates (91.1% vs. 43.4%, *p* < 0.001). The independent factors influencing in-hospital mortality in the immunocompromised patients included GI bleeding (OR 5.782, 95% CI 1.257–26.599, *p* = 0.024) and antiviral therapy ≥ 14 days (OR 0.232, 95% CI 0.059–0.911, *p* = 0.036). In the immunocompetent patients, age (OR 1.08, 95% CI 1.006–1.159, *p* = 0.032), GI bleeding (OR 10.036, 95% CI 1.183–85.133, *p* = 0.035), and time to diagnosis (OR 1.029, 95% CI 1.004–1.055, *p* = 0.021) were significant prognostic factors, with the age and diagnosis time cut-offs for survival being 70 years and 31.5 days, respectively. Conclusions: GI bleeding is the most common manifestation and prognostic factor in both groups. Early diagnosis and effective antiviral therapy can significantly reduce in-hospital mortality.

## 1. Introduction

Cytomegalovirus (CMV) is a ubiquitous and significant double-stranded herpetic virus, distinguished by its relatively high global seroprevalence and diverse clinical presentations [1]. In general, immunocompetent individuals typically exhibit mild and self-limited symptoms without involving end organs, contrasting sharply with the invasive and potentially fatal CMV disease seen in immunocompromised patients [2]. Studies across different disease populations underscore a significant clinical burden associated with CMV, irrespective of the host’s immune status. For example, CMV reactivation may increase both the colectomy rate and the risk of drug resistance in patients with inflammatory bowel disease (IBD) [3]; similarly, it can lead to a worse overall prognosis in patients receiving immunosuppressive management after solid organ or hematopoietic stem cell transplantation [4,5].

In immunocompromised individuals, the gastrointestinal (GI) tract is particularly susceptible to CMV infection, with potential involvement from the oral cavity to the anorectal region [6]. Notably, emerging studies have begun to illuminate CMV GI disease in patients who appear to be immunocompetent, bringing much-needed attention to this previously underrecognized group [7]. It is crucial to recognize that critical illness and various underlying diseases—such as diabetes mellitus, end-stage renal disease, and liver cirrhosis—may also contribute to immunodeficiency. While these conditions are not traditionally categorized as “immunocompromised” statuses, they can also compromise the immune system, thereby altering the clinical trajectory of CMV infections.

However, the current medical guidelines and literature still fall short in providing a detailed elaboration on clinical practices tailored for diverse immune statuses. This is particularly evident in aspects such as patient characteristics, clinical manifestations, treatment approaches, outcomes, risk factors, and prognostic factors. As a result, a more thorough characterization of the immunocompetent group afflicted with CMV GI diseases becomes critical in bridging this knowledge gap. The distinctions between immunocompetent and immunocompromised groups in the context of CMV GI diseases mostly remain ambiguous. While a number of case series have attempted to address these distinctions, their insights are often limited by small cohort sizes or less reliable histopathological diagnostic criteria [8,9,10]. Moreover, there is a scarcity of research focusing on the efficacy of antiviral treatments and the identification of outcome predictors specific to these distinct immune statuses.

In light of these limitations, this study aims to conduct a comprehensive comparison of immunocompromised and immunocompetent patients suffering from CMV GI diseases. Our focus extends to a detailed analysis of their clinical features, outcomes, and prognostic factors and the efficacies of antiviral therapies. Through this research, we aspire to contribute valuable insights and facilitate a deeper understanding of CMV GI diseases in varying immune contexts.

## 2. Materials and Methods

### 2.1. Ethics

This study adhered to the ethical guidelines of the 1975 Declaration of Helsinki and received approval from the Institutional Review Board (IRB) of the Chang Gung Medical Foundation (approval no. 202101234B0) for the period of 28 July 2021–27 July 2022. The study titled “Clinical presentations and outcome of cytomegalovirus, herpes simplex virus, Epstein-Barr virus, and Clostridioides difficile” was conducted as a retrospective analysis; hence, individual patient consent was waived.

### 2.2. Patient

Eligible patients with confirmed CMV GI diseases were retrospectively identified from a pathology database between January 2000 and July 2022. Diagnosis was established through positive immunohistochemistry (IHC) staining, which involved the use of a mouse monoclonal antibody blend (1:200 dilution, clone 8B1.2/1G5.2/2D4.2, Zeta Corporation, Arcadia, CA, USA), subsequently assessed with a BOND Polymer Refine Detection Kit (DS9800, Leica Biosystems, Wetzlar, Germany). Nuclear staining of either epithelial or mesenchymal cells in the GI tissue was considered a positive indicator, irrespective of the presence of CMV inclusion bodies.

### 2.3. Definition of Immune Status

Patients were classified as “immunocompromised” if they had confirmed primary immunodeficiency disorders or any acquired conditions. These conditions included human immunodeficiency virus (HIV) infection, exposure to chemotherapeutic agents or radiotherapy within the past six months, the use of immunosuppressants (including corticosteroids in oral or intravenous form, up to a dosage of 20 mg/day of prednisolone or its equivalent for more than two weeks), or a history of receiving solid organ or hematopoietic stem cell transplantation.

### 2.4. Data Extraction

We systematically reviewed electronic medical records to extract an array of relevant data. These data included demographic information (age at diagnosis, gender, source as either an inpatient or outpatient, time to diagnosis, duration of admission, and time of death or last follow-up), comorbidities, critical conditions preceding CMV diagnosis within a week (such as ventilator support and shock), necessity for intensive care unit (ICU) admission, baseline medication history, and primary symptoms presented. Endoscopic findings were categorized into three primary types: polypoid mass, ulcer, and inflammation (excluding cases with concurrent masses or ulcers). Histopathological reports were examined alongside a comprehensive range of laboratory findings, including white blood cell (WBC) count, segment, lymphocyte count, neutrophil-to-lymphocyte ratio (NLR), platelet count (Plt), hemoglobin (Hb), creatinine (Cr), aspartate aminotransferase (AST), alanine aminotransferase (ALT), bilirubin, albumin, C-reactive protein (CRP) levels, CMV pp65 antigenemia, and CMV viremia. The latter was assessed using the Light-Mix^®^ Kit human cytomegalovirus (TIB Molbiol, Berlin, Germany, cut-off: Cp 35, 226 bp segment on glycoprotein B gene) and the COBAS^®^ AmpliPrep/COBAS^®^ TaqMan^®^ CMV Test (Roche Diagnostics, Branchburg, NJ, USA, cut-off: 150 copies/mL), along with CMV serology results. Additionally, data on CMV disease-related operations, antiviral treatment details (drug type, administration routes, duration, and severe adverse events), complications (such as GI perforation), recurrence (defined as a new tissue-proven CMV infection in a patient previously diagnosed with CMV, following a virus-free interval of at least four weeks), and survival outcomes (in-hospital and overall mortality rates) were collected. The “time to diagnosis” was determined as the period from the first medical visit to the date of histopathological confirmation.

### 2.5. Statistical Analysis

Categorical data are presented as numbers and percentages, while numerical data are shown as mean ± standard deviation or median with IQR. Chi-square/Fisher’s exact tests and independent *t*-tests/Mann–Whitney *U* tests were used for categorical and continuous variables, respectively. Univariate and multivariate logistic regression models were employed to identify independent prognostic factors, with the results expressed as ORs, 95% CIs, and *p*-values. Survival analyses were conducted using Kaplan–Meier curves and log-rank tests, while ROC curve analyses and the Youden index method were used to determine the optimal cut-off values for significant continuous variables. A *p*-value < 0.05 was considered statistically significant. Statistical analyses were performed using Microsoft Excel and SPSS (Version 21.0., IBM Corp., Armonk, NY, USA).

## 3. Results

### 3.1. Patient Characteristics and Clinical Manifestations

A total of 401 patients diagnosed with CMV GI diseases were included in the final analysis, comprising 193 immunocompromised and 208 immunocompetent patients (Table 1). In terms of basic characteristics, the immunocompetent group was older, yet both groups exhibited similar gender ratios and patient sources. The immunocompetent patients more frequently presented with critical illness (such as ICU admission, respiratory failure, pneumonia, and acute kidney injury) and underlying comorbidities, including diabetes mellitus (DM), hypertension (HTN), past cerebral vascular accidents (CVAs), coronary artery disease (CAD), and ulcerative colitis (UC). Conversely, the immunocompromised group had a higher prevalence of malignancy, hematological diseases, and autoimmune disorders, likely linked to disease-related medication. Significant differences were noted in hemograms and select biochemistry items, as well as in the rates of CMV antigenemia/viremia between the groups; however, the number of available virology test results was limited. Coinfection rates with *Clostridioides difficile* and *Clostridium innocuum* in the CMV colitis subpopulation were comparable between both immune groups. GI bleeding emerged as the most common clinical manifestation, being more prevalent in the immunocompetent group (92.6% vs. 63.6%, *p* = 0.009). Regarding clinical outcomes, the immunocompetent group experienced longer hospital stays but lower in-hospital (18.9% vs. 32.2%, *p* = 0.034) and overall mortality rates (43.4% vs. 91.1%, *p* < 0.001) compared to the immunocompromised group. Notably, the duration of antiviral therapy was extended in the immunocompromised group. Antiviral treatments included intravenous ganciclovir (GCV), oral GCV, and oral valganciclovir (VGCV). 

### 3.2. Prognostic Factors

Using logistic regression models, various prognostic factors for in-hospital mortality were identified in each immunity group. In the immunocompromised group, 24 out of 55 examined factors were associated in the univariate analysis, with 2 remaining significant in the multivariate model: the presence of GI bleeding (OR 5.782, 95% CI 1.257–26.599; *p* = 0.024) and antiviral therapy for at least 14 days (OR 0.232, 95% CI 0.059–0.911; *p* = 0.036) (Table 2). In the immunocompetent group, 16 out of 54 factors were significant in the univariate analysis, with 3 being independently associated in the multivariate analysis: older age (OR 1.08, 95% CI 1.006–1.159; *p* = 0.032), presence of GI bleeding (OR 10.036, 95% CI 1.183–85.133; *p* = 0.035), and time to diagnosis (OR 1.029, 95% CI 1.004–1.055; *p* = 0.021) (Table 3).

### 3.3. Survival Curve Analysis and Efficacy of Antiviral Treatments

**Figure 1:**
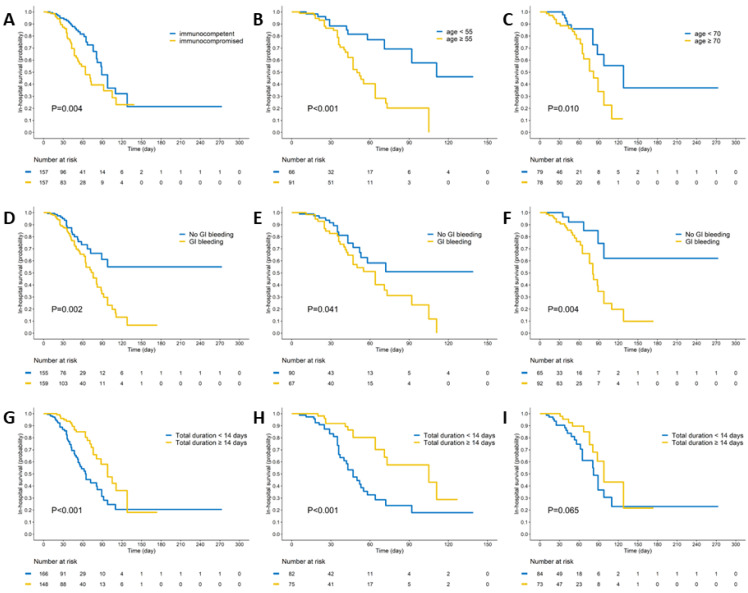
Kaplan–Meier survival curve analysis of CMV gastrointestinal (GI) diseases by immune status and clinical factors. (**A**) Immunocompromised patients exhibited significantly worse in-hospital mortality outcomes compared to immunocompetent patients (log-rank *p* = 0.004). (**B**) In the immunocompromised group, patients aged over 55 years demonstrated significantly poorer outcomes than their younger counterparts (log-rank *p* < 0.001). (**C**) In the immunocompetent group, patients aged over 70 years showed significantly worse outcomes compared to the younger patients (log-rank *p* = 0.01). (**D**) Patients with GI bleeding had significantly worse outcomes than those without GI bleeding (log-rank *p* = 0.002). (**E**,**F**) In both immunocompromised and immunocompetent groups, patients with GI bleeding experienced significantly worse outcomes (log-rank *p* = 0.041 and 0.004, respectively). (**G**) Patients who received an adequate duration of antiviral therapy (at least 14 days) had significantly better outcomes than those with a shorter therapy duration (log-rank *p* < 0.001). (**H**) In the immunocompromised group, patients receiving adequate (at least 14 days) antiviral therapy showed significantly better outcomes compared to those with a shorter therapy duration (log-rank *p* < 0.001). (**I**) In the immunocompetent group, patients receiving an adequate duration (at least 14 days) of antiviral therapy had similar outcomes to those with a shorter therapy duration (log-rank *p* = 0.065).

In the Kaplan–Meier survival curve analysis, the immunocompromised group exhibited significantly worse in-hospital survival compared to the immunocompetent group (log-rank *p* = 0.004). The patients presenting with GI bleeding (log-rank *p* = 0.002) or receiving antiviral therapy for less than 14 days (log-rank *p* < 0.001) also had poorer outcomes. A subgroup analysis revealed that, in both immunocompromised (log-rank *p* = 0.041) and immunocompetent (log-rank *p* = 0.004) groups, GI bleeding was associated with significantly worse survival outcomes. However, the impact of 14-day antiviral therapy on survival was only observed in the immunocompromised group (log-rank *p* < 0.001) (Figure 1).

### 3.4. AUROC Analysis of Continuous Variables

**Figure 2:**
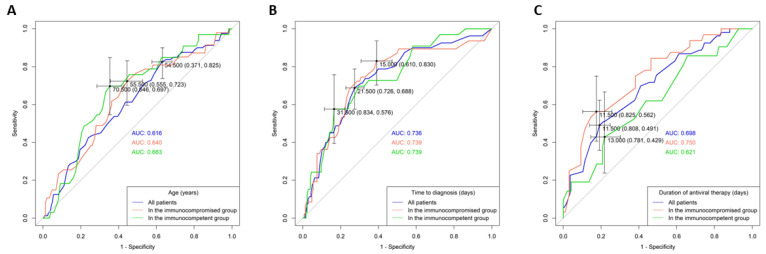
Receiver operating characteristic (ROC) curve and corresponding area under the curve (AUC) analysis for predicting in-hospital mortality in patients with different immune statuses. (**A**) Age (years) of all patients, the immunocompromised group, and the immunocompetent group. (**B**) Time to diagnosis (days) for all patients, the immunocompromised group, and the immunocompetent group. (**C**) Duration of antiviral therapy (days) for all patients, the immunocompromised group, and the immunocompetent group.

An AUROC analysis and the Youden index method were used to determine the cut-off values for the continuous variables associated with in-hospital mortality: age, duration of antiviral therapy, and time to diagnosis (Figure 2). For age, the cut-off values were set at 70.5 years for the immunocompetent group and 55.5 years for the immunocompromised group. Dichotomous division at 70 years for immunocompetent and 55 years for immunocompromised patients showed that older age groups (>70 years and >55 years) had significantly worse in-hospital survival outcomes (Figure 1B,C). The optimal cut-off values for the time-to-diagnosis period were 31.5 days for immunocompetent and 15 days for immunocompromised groups, and for the total antiviral therapy duration, they were 13 days for immunocompetent and 11 days for immunocompromised groups.

## 4. Discussion

CMV GI diseases affect individuals both with and without apparent immunodeficiency. Understanding the variations in manifestations between hosts with differing immune statuses is crucial for optimal clinical management. Our study, involving a large cohort proven through IHC staining, comprehensively compared these two immunity groups. The results revealed notable differences in patient characteristics, prognostic factors, survival outcomes, and the therapeutic benefits of antiviral treatments.

In this cohort, immunocompetent patients were not a minority. Despite not fitting the traditional definitions of immunodeficiency, they experienced various immunosuppressive conditions. Aging leads to “immunosenescence”, a progressive dysfunction of both innate and adaptive immune systems, whose mechanisms may involve reduced thymopoiesis and lymphocytic receptor diversity, telomere shortening with senescence, and malnutrition factors [11]. The elderly often face metabolic syndromes and systemic diseases (HTN, DM, old CVA, and CAD), exacerbating organ dysfunction. Additionally, critical illnesses like respiratory failure requiring mechanical ventilation, ICU admissions, and acute kidney injury were more common in this group, potentially contributing to immunosuppression. Diagnostically, symptomatology, virology, and endoscopic features between the groups were not distinctly different; the variations in the general laboratory indexes observed could be attributed to multiple etiologies. Yoon et al.’s study on an immunocompetent cohort also indicated that CMV GI disease typically develops in older patients with comorbidities, without a direct correlation between endoscopic features and clinical outcomes [10]. Other studies from Thailand also highlighted the prevalence of elderly and critical care (ICU) in immunocompetent patients [8,9]. Thus, “immunocompetent” patients with risk factors such as age, comorbidities, or critical conditions should not be overlooked as vulnerable to CMV GI diseases.

Regarding outcomes, the immunocompromised patients had significantly worse in-hospital survival, diverging from previous reports. One study reported higher six-month mortality among immunocompetent patients (39.0% vs. 22.0%, *p* = 0.047), while another found no difference in in-hospital survival between the groups (*p* = 0.65) [8,9]. These disparities could stem from differences in diagnostic criteria, definitions of immunocompromised status, patient features, disease severity, therapeutic approaches, and survival assessment endpoints.

Concerning the in-hospital mortality of CMV GI diseases, the existing literature predominantly discusses immunocompromised hosts or mixed-immunity cohorts. Our study contributes by identifying separate prognostic factors for both groups. GI bleeding was a universal negative prognostic factor, possibly indicating profound mucosal defects or impaired hemostasis, reflective of disease severity. Previous mixed-immunity cohort studies mentioned prognostic factors involving critical conditions, malnutrition, chemotherapy, and antiviral therapy [7,8,9].

For the immunocompetent group, older age was not only an immunosuppressive risk factor but also linked to worse prognosis, emphasizing the need for enhanced care for the elderly, especially those over 70. Interestingly, the impact of age was also significant in the survival curve analysis for the immunocompromised group (although not in the multivariate regression analysis), suggesting a younger age cut-off of 55 years. Furthermore, prolonged time to diagnosis was independently associated with worse in-hospital survival, presenting a clinical challenge, as CMV GI diseases are less suspected among “immunocompetent” patients. The mean time to diagnosis in our cohort was 19.9 days, shorter than the optimal cut-off of 31.5 days, yet some cases took up to 242 days to diagnose.

For the immunocompromised group, affirming antiviral intervention as a key prognostic factor was crucial. Given the pharmacological toxicity and variable efficacy, physicians often face uncertainty in prescribing antiviral treatments. While guidelines for non-GI CMV diseases are more available, the clinical evidence supporting antiviral treatment for GI diseases remains insufficient, except for in specific situations like inflammatory bowel disease and HIV. Our study revisits these controversies, elucidating the disparities in therapeutic benefits between the two immune groups. In the immunocompromised patients, an adequate antiviral therapy course of 11–14 days improved in-hospital survival, aligning with guidelines for other diseases, though not specified for GI diseases: for solid organ transplant patients, a minimum two-week treatment is recommended with viral eradication monitoring [12,13]; for post-hematopoietic stem cell transplant patients, standard treatment typically includes at least 14 days of GCV or VGCV [14,15]; HIV patients are advised to receive 2–4 weeks of GCV or VGCV until symptom resolution [16]. Despite these guidelines not covering all immunosuppressive etiologies in our cohort, a minimum 14-day course seems pragmatic. However, potential biases from heterogeneous underlying diseases and varying disease severity must be considered. Our study also did not explore antiviral drug resistance or emerging novel agents.

This study had limitations, including its retrospective design, which led to data heterogeneity (missing data, inconsistent symptom recording, and limited CMV virus status tests), and diverse care strategies over two decades. Further studies are needed to address these challenges. Nonetheless, as the currently largest IHC staining-based cohort of CMV GI diseases, our work adds comprehensive findings on demographics, prognosis assessments, and decision-making for antiviral interventions in patients with different immune statuses. The beneficial effects of treatment, more clearly elucidated, may reassure clinicians in selecting appropriate candidates and help build more therapeutic experiences.

## 5. Conclusions

In conclusion, older and multi-comorbid “immunocompetent” patients should be given greater diagnostic consideration. GI bleeding and delayed diagnosis are associated with worse prognosis, but adequate antiviral treatment can potentially improve survival outcomes, especially in immunocompromised individuals.

## Figures and Tables

**Table 1:** Comparisons of patient characteristics, diagnostic features, treatments, and outcomes of CMV GI diseases between two immune groups.

	Immunocompromised (N = 193)	Immunocompetent (N = 208)	Total (N = 401)	*p*-Value
General data				
Age (year) (mean ± SD)	54.6 ± 17.9	65.4 ± 17.3	60.2 ± 18.4	<0.001 *
Gender (male/female)	129/64	125/83	254/147	0.161
Outpatient/inpatient	38/155	51/157	89/312	0.245
Critical illness (N (%))				
Shock	37 (19.2)	50 (24)	87 (21.7)	0.237
Pneumonia	60 (31.1)	44 (21.2)	104 (25.9)	0.023 *
Intubation	26 (13.5)	49 (23.6)	75 (18.7)	0.010 *
Intensive care unit	36 (18.7)	60 (28.8)	96 (23.9)	0.017 *
AKI	30 (15.5)	50 (24)	80 (20)	0.033 *
Underlying diseases (N (%))				
Diabetes mellitus	31 (16.1)	85 (40.9)	116 (28.9)	<0.001 *
Hypertension	71 (36.8)	110 (52.9)	181 (45.1)	0.001 *
Old CVA	11 (5.7)	36 (17.3)	47 (11.7)	<0.001 *
COPD	9 (4.7)	9 (4.3)	18 (4.5)	0.871
CAD	17 (8.8)	33 (15.9)	50 (12.5)	0.033 *
Liver cirrhosis	7 (3.6)	10 (4.8)	17 (4.2)	0.558
ESRD	19 (9.8)	29 (13.9)	48 (12)	0.207
CKD	40 (20.7)	51 (24.5)	91 (22.7)	0.365
Hematology diseases	12 (6.2)	2 (1)	14 (3.5)	0.004 *
Crohn’s disease	5 (2.6)	7 (3.4)	12 (3)	0.773
Ulcerative colitis	14 (7.3)	28 (13.5)	42 (10.5)	0.043 *
HBV	22 (11.4)	16 (7.7)	38 (9.5)	0.205
HCV	10 (5.2)	7 (3.4)	17 (4.2)	0.367
Laboratory tests				
WBC (/uL)	7129.9 ± 4930.9	8696 ± 3835.6	7923.5 ± 4473.3	0.001 *
Segment (%)	75.1 ± 15.2	71.2 ± 13.9	73.1 ± 14.6	0.011 *
Lymphocyte (%)	14.5 ± 12.7	18.9 ± 12.2	16.7 ± 12.6	0.001 *
Hemoglobin (g/dL)	9.9 ± 2	10.6 ± 2.7	10.3 ± 2.4	0.005 *
Platelet (1000/uL)	193 ± 110.9	246.4 ± 119.9	220.1 ± 118.5	<0.001 *
Bilirubin (mg/dL)	1.5 ± 5.2	0.8 ± 1.1	1.2 ± 3.9	0.129
ALT (IU/L)	51.9 ± 144.1	25.4 ± 27.8	38.9 ± 105.4	0.016 *
Creatinine (mg/dL)	1.5 ± 2.6	1.9 ± 2.2	1.7 ± 2.4	0.068
Albumin (g/dL)	2.8 ± 0.7	3.1 ± 4	2.9 ± 2.8	0.306
CRP (mg/L)	66.2 ± 77.4	55.3 ± 61.7	60.5 ± 69.7	0.177
Virology tests (N-positive/N-tested (%))				
CMV-IgM	17/84 (20.2)	17/94 (18.1)	34/178 (19.1)	0.884
CMV-IgG	77/79 (97.5)	86/88 (97.7)	163/167 (97.6)	0.956
CMV-antigenemia	44/68 (64.7)	24/56 (42.9)	68/124 (54.8)	0.011 *
CMV-viremia	57/74 (77)	39/56 (69.6)	96/130 (73.8)	0.033 *
C. diff (colon)	7 (8.1)	17 (11.5)	24 (10.3)	0.416
CI (colon)	0 (0)	4 (2.7)	4 (1.7)	0.299
Symptoms (N (%))				
Fever	65 (33.7)	57 (27.4)	122 (30.4)	0.172
Abdominal pain	65 (33.7)	69 (33.2)	134 (33.4)	0.915
GI bleeding	75 (38.9)	108 (51.9)	183 (45.6)	0.009 *
Endoscopic findings (N (%))				
Polypoid mass	21 (10.9)	23 (11.1)	44 (11)	0.955
Inflammation	17 (8.8)	22 (10.6)	39 (9.7)	0.550
Ulcer	169 (87.6)	171 (82.2)	340 (84.8)	0.136
Treatment (N (%))				
Operation	14 (7.3)	11 (5.3)	25 (6.2)	0.416
Antiviral therapy				
IV and PO	37 (19.2)	32 (15.4)	69 (17.2)	0.316
IV or PO	128 (66.3)	119 (57.2)	247 (61.6)	0.061
IV duration (day)	14 (8, 18)	14 (7.25, 15)	14 (8, 16.5)	0.096
PO duration (day)	16 (10, 28)	15.5 (10, 22)	16 (10, 27.3)	0.063
Total duration (day)	17 (11, 34.5)	17 (12, 28)	17 (11, 28)	0.035 *
Total duration ≥ 14 days	90 (46.6)	87 (41.8)	177 (44.1)	0.333
Outcomes (N (%))				
Time to diagnosis (day)	19.2 ± 16.5	19.9 ± 21.3	19.6 ± 19.1	0.726
Admission time (day)	37.1 ± 27.3	44.7 ± 36.8	40.8 ± 32.6	0.039 *
Follow-up time (day)	855.9 ± 1346.4	905.2 ± 1343.7	881.5–1343.5	0.714
Perforation	6 (3.1)	5 (2.4)	11 (2.7)	0.666
Recurrence	10 (5.2)	10 (4.8)	20 (5)	0.573
In-hospital mortality	47 (24.4)	33 (15.9)	80 (20)	0.034 *
Overall mortality	92 (47.7)	63 (30.3)	155 (38.7)	<0.001 *

Abbreviations: AKI, acute kidney injury; ALT, alanine transaminase; CAD, coronary artery disease; C. diff, Clostridioides difficile infection; CI, *Clostridium innocuum* infection; CKD, chronic kidney disease; CMV, cytomegalovirus; COPD, chronic obstructive pulmonary disease; CRP, C-reactive protein; CVA, cerebral vascular accident; ESRD, end-stage renal disease; GI, gastrointestinal; HBV, hepatitis B; HCV, hepatitis C; IV, intravenous; PO, per os; SD, standard deviation; WBC, white blood cell; treatment durations are expressed as median (IQR (interquartile range)1, IQR3); *, *p*-value < 0.005.

**Table 2:** Prognostic factors associated with in-hospital mortality in immunocompromised and immunocompetent patients with CMV GI diseases.

	Immunocompromised						Immunocompetent					
	Univariate Analysis			Multivariate Analysis			Univariate Analysis			Multivariate Analysis		
	OR	95% CI	*p*-Value	OR	95% CI	*p*-Value	OR	95% CI	*p*-Value	OR	95% CI	*p*-Value
Age	1.029	1.008–1.050	0.007 *				1.036	1.008–1.066	0.012 *	1.08	1.006–1.159	0.032 *
Gender (male)	1.349	0.681–2.674	0.391				1.311	0.620–2.775	0.479			
Outpatient	0.064	0.009–0.481	0.008 *				0	0–	0.997			
Shock	10.676	4.763–23.929	<0.001 *				3.333	1.530–7.264	0.002 *			
Pneumonia	3.604	1.812–7.167	<0.001 *				6.877	3.084–15.334	<0.001 *			
Intubation	5.753	2.414–13.707	<0.001 *				5.574	2.536–12.251	<0.001 *			
Intensive care unit	7.054	3.218–15.464	<0.001 *				12.5	5.195–30.079	<0.001 *			
Diabetes mellitus	0.41	0.136–1.240	0.114				1.934	0.914–4.095	0.085			
Hypertension	1.742	0.893–3.398	0.103				1.691	0.784–3.647	0.180			
Old CVA	1.847	0.516–6.612	0.346				2.056	0.862–4.901	0.104			
COPD	1.591	0.382–6.626	0.524				0	0–	0.999			
CAD	2.38	0.851–6.655	0.098				2.359	0.980–5.682	0.056			
Liver cirrhosis	0.507	0.059–4.324	0.535				0.576	0.071–4.759	0.607			
ESRD	0.554	0.154–1.992	0.366				1.872	0.727–4.825	0.194			
CKD	1.685	0.785–3.616	0.180				0.802	0.325–1.975	0.631			
AKI	2.901	1.284–6.555	0.010 *				2.845	1.302–6.216	0.009 *			
Malignancy	2.666	1.355–5.244	0.005 *				0.775	0.217–2.773	0.695			
Chemotherapy	1.717	0.872–3.382	0.118				na	na	na			
Radiotherapy	2.211	1.091–4.483	0.028 *				na	na	na			
Transplant	0.706	0.225–2.213	0.550				0	0–	1			
Hematology disease	1.605	0.461–5.59	0.458				5.437	0.332–89.179	0.235			
Autoimmune/PID	0.75	0.265–2.123	0.588				5.581	0.758–41.108	0.092			
Crohn’s disease	0	0–	0.999				0	0–	0.999			
Ulcerative colitis	0.496	0.107–2.302	0.371				0.171	0.022–1.307	0.089			
HIV	0.216	0.063–0.74	0.015 *				na	na	na			
HBV	0.456	0.129–1.615	0.223				1.246	0.335–4.639	0.743			
HCV	0.331	0.041–2.683	0.300				0.88	0.102–7.56	0.907			
Corticosteroids	2.484	1.173–5.261	0.017 *				1.398	0.523–3.74	0.504			
Immunosuppressant	0.448	0.186–1.081	0.074				0	0–	1			
NLR	1.042	1.022–1.063	<0.001 *				1.08	1.044–1.118	<0.001 *			
Hemoglobin	0.775	0.651–0.924	0.004 *				0.809	0.664–0.986	0.036 *			
Platelet	0.993	0.989–0.997	<0.001 *				0.992	0.988–0.996	<0.001 *			
Bilirubin	1.05	0.97–1.137	0.226				1.046	0.715–1.528	0.818			
ALT	1.007	1.001–1.013	0.023 *				1.005	0.992–1.017	0.458			
Creatinine	1.108	0.961–1.278	0.159				1	0.837–1.195	1			
Albumin	0.31	0.168–0.572	<0.001 *				0.217	0.089–0.531	0.001 *			
CRP	1.009	1.004–1.014	<0.001 *				1.01	1.004–1.016	0.001 *			
Fever	1.663	0.845–3.27	0.141				3.076	1.428–6.624	0.004 *			
Abdominal pain	1.022	0.51–2.046	0.952				0.719	0.314–1.644	0.434			
GI bleeding	4.491	2.232–9.038	<0.001 *	5.782	1.257–26.599	0.024 *	6.65	2.454–18.022	<0.001 *	10.036	1.183–85.133	0.035 *
Lesion—polypoid	0.485	0.136–1.725	0.264				0	0–	0.998			
Lesion—inflammation	0.388	0.085–1.764	0.220				1.203	0.38–3.813	0.753			
Lesion—ulcer	3.992	0.902–17.663	0.068				1.685	0.554–5.122	0.358			
Operation	2.524	0.828–7.694	0.103				3.31	0.911–12.028	0.069			
Any antiviral therapy	0.979	0.489–1.96	0.952				1.375	0.637–2.968	0.417			
Therapy duration ≥ 14 days	0.391	0.193–0.790	0.009 *	0.232	0.059–0.911	0.036 *	0.762	0.353–1.645	0.489			
Time-to-diagnosis period	1.044	1.021–1.068	<0.001 *				1.023	1.003–1.043	0.021 *	1.029	1.004–1.055	0.021 *

Abbreviations: AKI, acute kidney injury; ALT, alanine transaminase; CAD, coronary artery disease; CI, confidence interval; CKD, chronic kidney disease; CMV, cytomegalovirus; COPD, chronic obstructive pulmonary disease; CRP, C-reactive protein; CVA, cerebral vascular accident; ESRD, end-stage renal disease; GI, gastrointestinal; HBV, hepatitis B; HCV, hepatitis C; HIV, human immunodeficiency virus; NLR, neutrophil–lymphocyte ratio; OR, odds ratio; PID, primary immunodeficiency disorder; *, *p*-value <0.005.

**Table 3:** Prognostic factors associated with in-hospital mortality in immunocompetent patients with CMV GI diseases.

	Univariate Analysis			Multivariate Analysis		
	OR	95% CI	*p*-Value	OR	95% CI	*p*-Value
Age	1.036	1.008–1.066	0.012 *	1.08	1.006–1.159	0.032 *
Gender (male)	1.311	0.620–2.775	0.479			
Outpatient	0	0-	0.997			
Shock	3.333	1.530–7.264	0.002 *			
Pneumonia	6.877	3.084–15.334	<0.001 *			
Intubation	5.574	2.536–12.251	<0.001 *			
ICU	12.5	5.195–30.079	<0.001 *			
DM	1.934	0.914–4.095	0.085			
HTN	1.691	0.784–3.647	0.180			
Old CVA	2.056	0.862–4.901	0.104			
COPD	0	0-	0.999			
CAD	2.359	0.980–5.682	0.056			
LC	0.576	0.071–4.759	0.607			
ESRD	1.872	0.727–4.825	0.194			
CKD	0.802	0.325–1.975	0.631			
AKI	2.845	1.302–6.216	0.009 *			
Malignancy	0.775	0.217–2.773	0.695			
Chemotherapy	na	na	na			
Radiotherapy	na	na	na			
Transplant	0	0-	1			
Hematology disease	5.437	0.332–89.179	0.235			
Autoimmune/PID	5.581	0.758–41.108	0.092			
Crohn’s disease	0	0-	0.999			
Ulcerative colitis	0.171	0.022–1.307	0.089			
HIV	na	na	na			
HBV	1.246	0.335–4.639	0.743			
HCV	0.88	0.102–7.56	0.907			
Corticosteroids	1.398	0.523–3.74	0.504			
Immunosuppressant	0	0-	1			
NLR	1.08	1.044–1.118	<0.001 *			
Hemoglobin	0.809	0.664–0.986	0.036 *			
Platelet	0.992	0.988–0.996	<0.001 *			
Bilirubin	1.046	0.715–1.528	0.818			
ALT	1.005	0.992–1.017	0.458			
Creatinine	1	0.837–1.195	1			
Albumin	0.217	0.089–0.531	0.001 *			
CRP	1.01	1.004–1.016	0.001 *			
Fever	3.076	1.428–6.624	0.004 *			
Abdominal pain	0.719	0.314–1.644	0.434			
GI bleeding	6.65	2.454–18.022	<0.001 *	10.036	1.183–85.133	0.035 *
Lesion—polypoid	0	0-	0.998			
Lesion—inflammation	1.203	0.38–3.813	0.753			
Lesion—ulcer	1.685	0.554–5.122	0.358			
Operation	3.31	0.911–12.028	0.069			
Any antiviral therapy	1.375	0.637–2.968	0.417			
Therapy duration ≥ 14 days	0.762	0.353–1.645	0.489			
Time-to-diagnosis period	1.023	1.003–1.043	0.021 *	1.029	1.004–1.055	0.021 *

Abbreviations: AKI, acute kidney injury; ALT, alanine transaminase; CAD, coronary artery disease; CI, confidence interval; CKD, chronic kidney disease; CMV, cytomegalovirus; COPD, chronic obstructive pulmonary disease; CRP, C-reactive protein; CVA, cerebral vascular accident; ESRD, end-stage renal disease; GI, gastrointestinal; HBV, hepatitis B; HCV, hepatitis C; HIV, human immunodeficiency virus; na, not available; NLR, neutrophil–lymphocyte ratio; OR, odds ratio; PID, primary immunodeficiency disorder; *, *p*-value <0.005.

## Data Availability

The data presented in this study are available on request from the corresponding author.
